# Intraspecific variation and phylogeography of the millipede model organism, the Black Pill Millipede *Glomerismarginata* (Villers, 1789) (Diplopoda, Glomerida, Glomeridae)

**DOI:** 10.3897/zookeys.741.21917

**Published:** 2018-03-07

**Authors:** Hans S. Reip, Thomas Wesener

**Affiliations:** 1 Senckenberg Museum für Naturkunde Görlitz, Am Museum 1, 02826 Görlitz, Germany Senckenberg Museum für Naturkunde Görlitz Görlitz Germany; 2 Zoologisches Forschungsmuseum Alexander Koenig, Leibniz Institute for Animal Biodiversity, Adenauerallee 160, D-53113 Bonn, Germany Leibniz Institute for Animal Biodiversity Bonn Germany

**Keywords:** biogeographic regions, COI, Europe, haplotype analysis, haplotype richness estimation

## Abstract

The Black Pill Millipede, *Glomerismarginata*, is the best studied millipede species and a model organism for Diplopoda. *Glomerismarginata* is widespread, with numerous colour morphs occurring across its range, especially in the south. This study investigates whether colour morphs might represent cryptic species as well as the haplotype diversity and biogeography of *G.marginata*. The results of the COI barcoding fragment analysis include 97 *G.marginata*, as well as 21 specimens from seven potentially related species: *G.intermedia* Latzel, 1884, *G.klugii* Brandt, 1833 (*G.undulata* C.L. Koch, 1844), *G.connexa* Koch, 1847, *G.hexasticha* Brandt, 1833, *G.maerens* Attems, 1927, *G.annulata* Brandt, 1833 and *G.apuana* Verhoeff, 1911. The majority of the barcoding data was obtained through the German Barcode of Life project (GBOL). Interspecifically, *G.marginata* is separated from its congeners by a minimum uncorrected genetic distance of 12.9 %, confirming its monophyly. Uncorrected intraspecific distances of *G.marginata* are comparable to those of other widespread *Glomeris* species, varying between 0–4.7%, with the largest genetic distances (>2.5 %) found at the Mediterranean coast. 97 sampled specimens of *G.marginata* yielded 47 different haplotypes, with identical haplotypes occurring at large distances from one another, and different haplotypes being present in populations occurring in close proximity. The highest number of haplotypes was found in the best-sampled area, western Germany. The English haplotype is identical to northern Spain; specimens from southern Spain are closer to French Mediterranean specimens. Analyses (CHAO1) show that approximately 400 different haplotypes can be expected in *G.marginata*. To cover all haplotypes, it is projected that up to 6,000 specimens would need to be sequenced, highlighting the impossibility of covering the whole genetic diversity in barcoding attempts of immobile soil arthropod species.

## Introduction

In recent decades the Black Pill Millipede, *G.marginata* (Glomerida, Glomeridae) has become a model organism of the Diplopoda. The Black Pill Millipede is morphologically the best studied species of the millipedes (see examples in Koch 2015). Studies include muscle supercontraction (Candia Carnevali and Valvassori 1982), the digestive tract (Schlüter 1980, Martin and Kirkham 1989), the tracheal system (Verhoeff 1895, Wernitzsch 1910, Hilken 1998, Hilken et al. 2015), the Malpighian tubule system (Johnson and Riegel 1977a, 1977b), the postgonopodial glands (Juberthie-Jupeau 1978) and sensorial system (Sahli 1966, Seifert 1966, Müller and Sombke 2015). Additionally, numerous studies on the chemical composition of the integument of millipedes are based on *G.marginata* (Ansenne et al. 1990, Compère et al. 1996, Makarov 2015).

After discovering a new chemical compound in *G.marginata* (Glomerin: Schildknecht et al. 1966), further studies on the defensive secretions of *G.marginata* were conducted by several authors (Meinwald et al. 1966, Schildknecht et al. 1967, Schildknecht and Wenneis 1967, Carrell 1984). For a long time (see Shear et al. 2011) *G.marginata* was the only animal species known to sequester quinazolinone alkaloids. *Glomerismarginata* is the only millipede species in which the embryonic and postembryonic development is thoroughly known (Dohle 1964, Juberthie-Jupeau 1967, Enghoff et al. 1993, Janssen 2004, Prpic 2004).

The unusual mating behaviour of pill millipedes (involving the sperm ejaculation on a piece of soil before the transfer to the female) was studied extensively in the Black Pill Millipede (e.g., Haacker 1964). The ecology of the species was also the subject of numerous studies (for single aspects e.g., Nicholson et al. 1966, Van der Drift 1975, David and Gillon 2002, Rawlins et al. 2006; for the role in species communities e.g., Dunger and Steinmetzger 1981 and Voigtländer 2011). The Black Pill Millipede was also the first myriapod species in which the pheromone producing postgonopodial glands were studied (Juberthie-Jupeau 1967).

*Glomerismarginata* is commonly included in arthropod phylogenetic analyses (e.g., Regier 2001, 2005). The Black Pill Millipede is the only species of the Diplopoda in which gene expressions of different genes, including Hox genes, were widely researched (e.g., Prpic and Tautz 2003, Prpic 2005, Prpic et al. 2005, Janssen et al. 2006, Janssen and Damen 2006). Recently, the embryonic expression of Wnt genes was studied for the first time in myriapods (Janssen and Posnien 2014) in this species. Additionally, the embryonic development, especially the embryonic development of the segmentation inside the Myriapoda, is currently nowhere as well known as in *G.marginata* (Enghoff et al. 1993, Janssen 2011, 2013, Fusco and Minelli 2013, Minelli and Fusco 2013, Minelli 2015). The same applies to the neurogenesis (Dove 2003).

Despite the high importance of *G.marginata* for general studies of millipedes, and arthropod segmentation patterns in general, little to no taxonomic studies or population genetic studies of the species were conducted in recent decades. Recent genetic studies in congeneric pill millipedes allowed the detection of several synonymies as well as cryptic species, and clarified the taxonomic status of several *Glomeris* species (Hoess and Scholl 1999, 2001, Wesener 2015a, 2015b, Conrad and Wesener 2016).

The lack of taxonomic studies in *G.marginata* is even more surprising considering the unusual wide distribution of the species (Kime and Enghoff 2011). *Glomerismarginata* is the only pill millipede reaching northern Europe. Its southernmost distribution is the south-eastern part of Spain alongside the southern border of the Pyrenees. The area of distribution of *G.marginata* covers France, England/Wales and Ireland, the whole of Germany except southern Bavaria and Saxony and extends north through Denmark to southern Sweden/Norway (Hoess 1999, Kime and Enghoff 2011: p. 104). *Glomerismarginata* is the most common pill millipede species in Germany (Reip et al. 2016).

**Figure 1.**
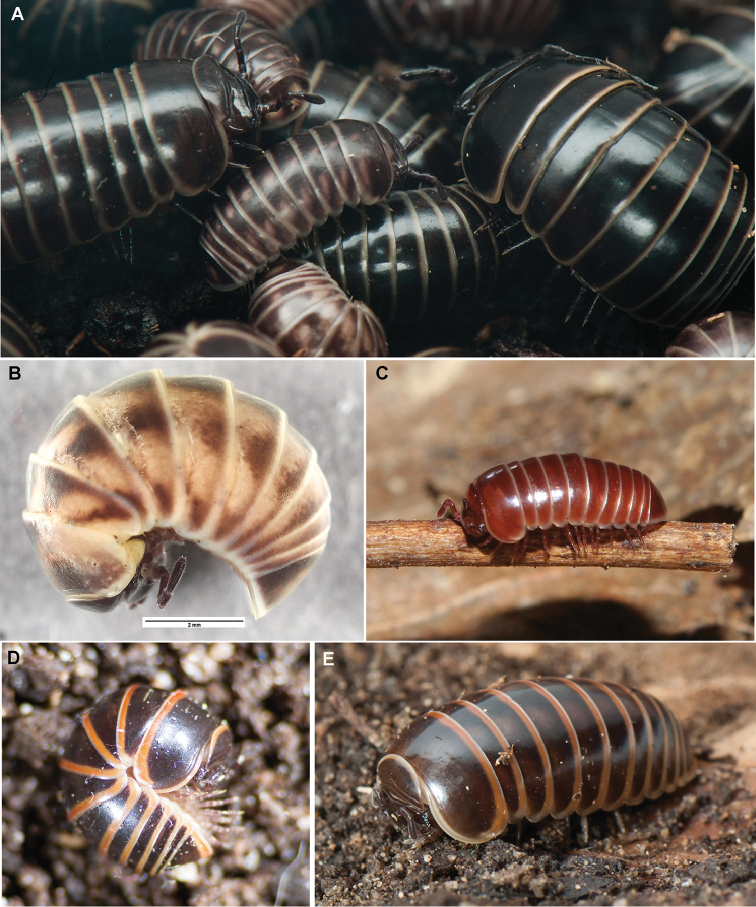
*Glomerismarginata* (Villers, 1789) colour morphs. **A** main coloration form, center immature specimens showing the perplexa colour pattern; Germany, Landskrone **B** strongly lightened adult perplexa pattern, France, Pays de la Loire **C** red mutant, Germany, Bonn **D** strongly red-banded form, from France, Montauroux **E** more weakly red-brown banded from, France, same population as D. **A, D, E** photographed by Jan Philip Oeyen **B** by ZFMK**C** by Dennis Rödder.

**Figure 2.**
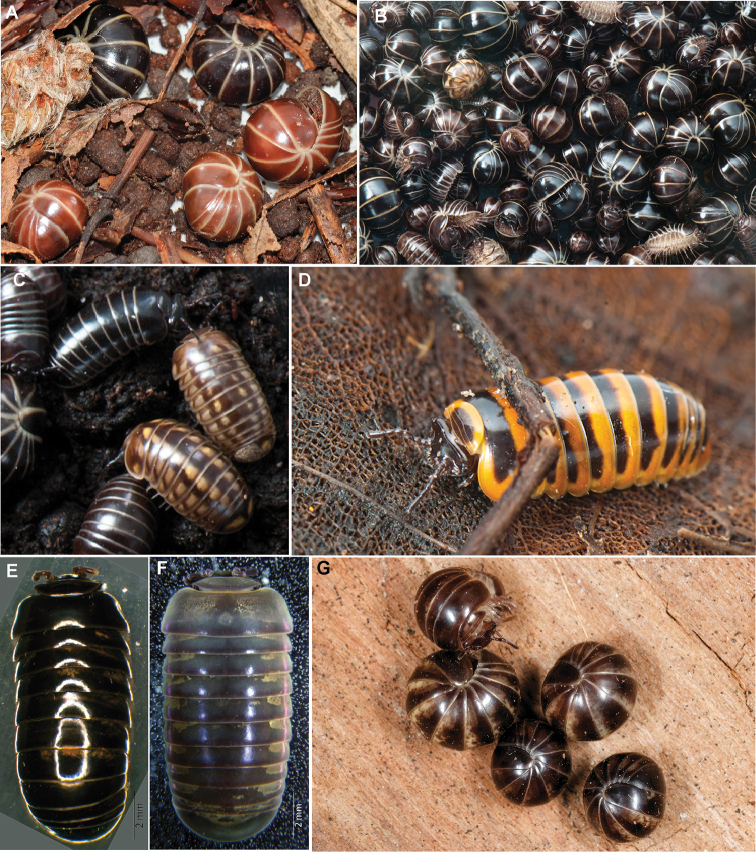
**A***G.marginata*, brown and black form occurring in sympatry, Germany, Rügen, 2016. **B–G** Similar coloured species of *Glomeris* analyzed in this study **B***G.marginata*, with a single specimen of *G.intermedia* in the upper left part, Germany, Landskrone, 2015 **C***G.intermedia* Latzel, 1884, with sympatric *G.marginata*, Germany, Landskrone, 2015 **D***G.annulata* Brandt, 1833, France, Gard, Courry, 2015 **E***G.cf.lugubris* Attems, 1952, Spain, Cádiz/ Sierra de Grazalema, 2008, preserved specimen**F***G.cf.maerens* Attems, 1927, Spain, Aragón/Teruel, 2010, preserved specimen **G***G.maerens*, Spain, Tarragona/Montsià, 2017; **B–D** photographed by Jan Philip Oeyen.

While adult *G.marginata* normally can be easily distinguished from their congeners by their shiny completely black-brown colour with brightly coloured creamy-white tergal margins (see Schubart 1934: 32, Hoess 2000, Figure 1A), several unusual specimens (grey or reddish, with prominent white marks, or with orange or reddish margins, see Figures 1B–E, 2A, B), currently interpreted as colour morphs, are often encountered. Such unusual specimens resemble other species of the genus, such as *G.intermedia* Latzel, 1884 (Figure 2B, C), which shares a similar, but more western, distribution pattern than *G.marginata*, or *G.annulata* Koch, 1847 (Figure 2D), a local endemic in southern France (Hoess 2000, Kime and Enghoff 2011). Two other local endemic species, *G.apuana* Verhoeff, 1911 (see Wesener 2015b) and *G.maerens* Attems, 1927 (Figures 2E–G) not only occur in areas directly bordering the known distribution of *G.marginata*, but also show a similar colour pattern. Furthermore, the species *G.klugii* Brandt, 1833 / *G.undulata* C.L. Koch, 1844 and *G.connexa* Koch, 1847 sometimes also appear in dark-brown colour forms.

In this work, it is tested whether *G.marginata* and its different colour variants form a monophyletic taxon based on barcoding mt-DNA COI data. The phylogeographic relationship and the possible origin of the species are also ascertained. Finally, the relationship of the Black Pill Millipede to the other, similar coloured congeneric species, *G.annulata*, *G.apuana*, and *G.maerens* is clarified.

## Material and methods

### Selection of specimens

Based on the project German Barcoding of Life (GBOL, http://www.bolgermany.de), 80 specimens of *G.marginata* from different locations were selected from the collection of the ZFMK (Zoologisches Forschungsmuseum Alexander Koenig, Bonn, Germany). All specimens of *G.annulata*, *G.apuana* and the *G.maerens* species-group came from the collection of the ZFMK, while the two specimens of *G.hexasticha* were collected by the first author. Six additional COI-sequences of *G.marginata* were obtained from former projects of the authors (see Spelda et al. 2011 and Wesener et al. 2010). These sequences are available from GenBank (see Table 1 for accession numbers). Also, the COI-sequences of the outgroup species *G.intermedia*, *G.klugii*/*undulata*, and *G.connexa* were obtained from the work of Spelda et al. (2011). An additional 11 French COI-sequences of *G.marginata* were available in BOLD (downloadable at the Public Data Portal, http://www.boldsystem.org, see Table 1 for BOLD-numbers) by end of November 2015. In total 97 COI-sequences of *G.marginata* and 21 of the seven outgroup species were obtained for this study (93 newly sequenced, 14 from GenBank and 11 from BOLD).

**Table 1.** Analysed specimens, voucher and Genbank code, collection locality and bioregion (see Table 2).

**SpecimenID**	**Voucher #**	**GenBank #**		**Lat./Lon.**	**BioRegion**
*Glomeris marginata*					
G.mar.01	GBOL33714	MG892112	Germany, Sachsen-Anhalt, Wernigerode, Königshütte	N51.743, E10.767	DE.MGSO
G.mar.02	ZFMK100409275	MG892115	Germany, Sachsen-Anhalt, Wernigerode, Königshütte	N51.744, E10.767	DE.MGSO
G.mar.03	ZFMK1634	MG892119	Germany, Niedersachsen, Goslar, Bockswiese	N51.841, E10.326	DE.MGSO
G.mar.04	ZFMK1909	MG892123	Germany, Thüringen, Saale-Holzland-Kreis, Schöngleina	N50.895, E11.753	DE.MGSO
G.mar.05	ZFMK19531	MG892146	Germany, Thüringen, Saale-Holzland-Kreis, Schöngleina	N50.895, E11.753	DE.MGSO
G.mar.06	ZFMK2503693	MG892153	Germany, Thüringen, Jena	N50.919, E11.548	DE.MGSO
G.mar.07	ZFMK2503694	MG892154	Germany, Thüringen, Jena	N50.919, E11.548	DE.MGSO
G.mar.08	ZFMK2542470	MG892173	Germany, Thüringen, Stadtroda, Hermsdorf	N50.892, E11.821	DE.MGSO
G.mar.09	ZFMK2542471	MG892174	Germany, Thüringen, Stadtroda, Hermsdorf	N50.892, E11.821	DE.MGSO
G.mar.10	ZFMK2542541	MG892175	Germany, Sachsen-Anhalt, Burgenland, Bad Kösen	N51.133, E11.749	DE.MGSO
G.mar.11	ZFMK2542542	MG892176	Germany, Sachsen-Anhalt, Burgenland, Bad Kösen	N51.133, E11.749	DE.MGSO
G.mar.12	ZFMK18967	MG892124	Germany, Nordrhein-Westfalen, Bonn, Wachtberg	N50.663, E7.103	DE.MGSW
G.mar.13	ZFMK18987	MG892126	Germany, Nordrhein-Westfalen, Königswinter	N50.666, E7.216	DE.MGSW
G.mar.14	ZFMK18988	MG892127	Germany, Nordrhein-Westfalen, Quirrenbach	N50.687, E7.300	DE.MGSW
G.mar.15	ZFMK18991	MG892128	Germany, Nordrhein-Westfalen, Hennef, Blankenberg	N50.767, E7.367	DE.MGSW
G.mar.16	ZFMK19003	MG892129	Germany, Nordrhein-Westfalen, Hagen-Holthausen	N51.361, E7.550	DE.MGSW
G.mar.17	ZFMK19005	MG892130	Germany, Nordrhein-Westfalen, Hagen-Holthausen	N51.361, E7.550	DE.MGSW
G.mar.18	ZFMK19029	MG892132	Germany, Nordrhein-Westfalen, Bad Münstereifel	N50.560, E6.808	DE.MGSW
G.mar.19	ZFMK19031	MG892133	Germany, Nordrhein-Westfalen, Wuppertal, Krutscheid	N51.230, E7.054	DE.MGSW
G.mar.20	ZFMK19044	MG892136	Germany, Nordrhein-Westfalen, Siegburg	N50.803, E7.242	DE.MGSW
G.mar.21	ZFMK19045	MG892137	Germany, Nordrhein-Westfalen, Hattingen, Felderbachtal	N51.359, E7.170	DE.MGSW
G.mar.22	ZFMK19046	MG892138	Germany, Nordrhein-Westfalen, Wuppertal, Krutscheid	N51.230, E7.054	DE.MGSW
G.mar.23	ZFMK19047	MG892139	Germany, Nordrhein-Westfalen, Bonn, Oberkassel	N50.714, E7.177	DE.MGSW
G.mar.24	ZFMK19048	MG892140	Germany, Nordrhein-Westfalen, Bonn, Röttgen	N50.672, E7.047	DE.MGSW
G.mar.25	ZFMK19049	MG892141	Germany, Nordrhein-Westfalen, Wuppertal, NSG Im Hölken	N51.291, E7.252	DE.MGSW
G.mar.26	ZFMK19051	MG892142	Germany, Rheinland-Pfalz, Ahrweiler, Heppingen	N50.551, E7.172	DE.MGSW
G.mar.27	ZFMK19054	MG892143	Germany, Rheinland-Pfalz, Niederzissen, Bausenberg	N50.465, E7.223	DE.MGSW
G.mar.28	ZFMK19057	MG892144	Germany, Nordrhein-Westfalen, Hagen-Holthausen	N51.361, E7.550	DE.MGSW
G.mar.29	ZFMK19539	MG892147	Germany, Nordrhein-Westfalen, Heimbach, Meuchelberg	N50.632, E6.473	DE.MGSW
G.mar.30	ZFMK19550	MG892148	Germany, Nordrhein-Westfalen, Neunkirchen, Hellerberg	N50.780, E8.009	DE.MGSW
G.mar.31	ZFMK19555	MG892149	Germany, Nordrhein-Westfalen, Neunkirchen, Hellerberg	N50.780, E8.009	DE.MGSW
G.mar.32	ZFMK19558	MG892150	Germany, Rheinland-Pfalz, Altenkirchen, Giesenhausen	N50.709, E7.713	DE.MGSW
G.mar.33	ZFMK19560	MG892151	Germany, Rheinland-Pfalz, Altenkirchen, Giesenhausen	N50.709, E7.713	DE.MGSW
G.mar.34	ZFMK19561	MG892152	Germany, Rheinland-Pfalz, Altenkirchen, Giesenhausen	N50.709, E7.713	DE.MGSW
G.mar.35	ZFMK2516208	MG892156	Germany, Nordrhein-Westfalen, Bad Honnef, Kasselbachtal	N50.625, E7.194	DE.MGSW
G.mar.36	ZFMK2516209	MG892157	Germany, Nordrhein-Westfalen, Bad Honnef, Kasselbachtal	N50.625, E7.194	DE.MGSW
G.mar.37	ZFMK2557907	MG892181	Germany, Hessen, Eschwege, Wanfried	N51.182, E10.221	DE.MGSW
G.mar.38	ZFMK2557908	MG892182	Germany, Hessen, Eschwege, Wanfried	N51.182, E10.221	DE.MGSW
G.mar.39	ZFMK100409283	MG892116	Germany, Schleswig-Holstein, Segeberg, Bockhorn	N53.919, E10.098	DE.NDTO
G.mar.40	ZFMK2538190	MG892171	Germany, Schleswig-Holstein, Weissenhaus	N54.303, E10.756	DE.NDTO
G.mar.41	ZFMK2538253	MG892172	Germany, Brandenburg, Pfingstberg, Schorfheide	N53.124, E13.884	DE.NDTO
G.mar.42	ZFMK2553394	MG892177	Germany, Mecklenburg-Vorpommern, Schwerin, Schweriner Innensee	N53.653, E11.437	DE.NDTO
G.mar.43	ZFMK2553395	MG892178	Germany, Mecklenburg-Vorpommern, Schwerin, Schweriner Innensee	N53.653, E11.437	DE.NDTO
G.mar.44	ZFMK2553405	MG892179	Germany, Brandenburg, Pritzwalk, Putlitz	N53.279, E12.077	DE.NDTO
G.mar.45	ZFMK100409272	MG892114	Germany, Niedersachsen, Soltau-Fallingbostel, Hebenbrock	N52.960, E9.893	DE.NDTW
G.mar.46	ZFMK19472	MG892145	Germany, Nordrhein-Westfalen, Bochum, Botanical Garden	N51.442, E7.267	DE.NDTW
G.mar.47	ZFMK100409123	MG892113	Germany, Bayern, Main-Spessart, Karlstadt	N49.983, E9.768	DE.SSL
G.mar.48	ZFMK100409296	MG892117	Germany, Bayern, Würzburg, Erlabrunn	N49.864, E9.857	DE.SSL
G.mar.49	ZFMK1861	MG892120	Spain, La Rioja, Navarrete	N42.430, W2.562	ES.CC
G.mar.50	ZFMK1863	MG892121	Spain, Navarra, Etxalar	N43.234, W1.638	ES.CC
G.mar.51	ZFMK1893	MG892122	Spain, Navarra, Etxalar	N43.234, W1.638	ES.CC
G.mar.52	ZFMK2517202	MG892159	Spain, Cataluña, Tarragona, Farena	N41.315, E1.104	ES.PYRS
G.mar.53	BGI12GEU183	MG892183	France, Auvergne-Rhône-Alpes, Isere, Grenoble	N45.273, E5.766	FR.ALP
G.mar.54	ZFMK2517217	MG892168	France, Auvergne-Rhône-Alpes, Isere, Oisans	N45.071, E6.008	FR.ALP
G.mar.55	ZFMK2553457	MG892180	France, Pays de la Loire, Mayenne, Saint-Pierre-sur-Orthe	N48.201, E0.171	FR.ATLN
G.mar.56	ZFMKTW163	MG931019	France, Pays de la Loire, Mayenne, Saint-Martin-de-Connée	N48.230, W0.242	FR.ATLN
G.mar.57	ZFMKTW164	MG931020	France, Centre-Val de Loire, Chinon, Rigny-Ussé	N47.261, E0.326	FR.ATLN
G.mar.58	ZFMK100410157	MG892118	France, Alsace, Haut-Rhin, Col du Hundsruck, Thann	N47.812, E7.065	FR.CONN
G.mar.59	ZFMK18996	MG931021	Luxemburg, , Schengen	N49.461, E6.364	FR.CONN
G.mar.60	ZFMK2517315	MG892169	France, Bourgogne-Franche-Comté, Luxeuil-les-Bains	N47.859, E6.404	FR.CONN
G.mar.61	ZFMK2517322	MG8921701	France, Elsas, Ballons des Vosges, Faucogney-et-la-Mer	N47.839, E6.667	FR.CONN
G.mar.62	ZFMKTW161	MG892184	France, Elsas, Ballons des Vosges, Faucogney-et-la-Mer	N47.839, E6.667	FR.CONN
G.mar.63	ZFMKTW162	MG892185	France, Elsas, Ballon d’Alcas, Sewen	N47.817, E6.874	FR.CONN
G.mar.64	ZFMK2517209	MG892160	France, Haute-Vienne-Corrèze-Creuse, Limousin, Correze	N45.235, E1.545	FR.CONS
G.mar.65	ZFMK18977	MG892125	France, Provence-Alpes-Côte d’Azur, Bédoin, Vaucluse	N44.114, E5.241	FR.MED
G.mar.66	ZFMK19021	MG892131	France, Provence-Alpes-Côte d’Azur, Bédoin, Vaucluse	N44.114, E5.241	FR.MED
G.mar.67	ZFMK19037	MG892134	France, Provence-Alpes-Côte d’Azur, Bédoin, Vaucluse	N44.114, E5.241	FR.MED
G.mar.68	ZFMK2516203	MG892155	France, Rhône-Alpes, Drôme, La Bégude-de-Mazenc	N44.551, E4.949	FR.MED
G.mar.69	ZFMK2517213	MG892164	France, Provence-Alpes-Côte d’Azur, Var	N43.494, E5.521	FR.MED
G.mar.70	ZFMK2517214	MG892165	France, Provence-Alpes-Côte d’Azur, Var	N43.464, E5.800	FR.MED
G.mar.71	ZFMK2517215	MG892166	France, Provence-Alpes-Côte d’Azur, Pierrefeu	N43.232, E6.234	FR.MED
G.mar.72	ZFMK2517216	MG892167	France, Provence-Alpes-Côte d’Azur, Lantosque	N43.974, E7.311	FR.MED
G.mar.73	ZFMKTW102	MG892186	France, Languedoc-Roussillon-Midi-Pyrénées, Courry	N44.297, E4.152	FR.MED
G.mar.74	ZFMKTW165	MG892187	France, Alpes-Côte d’Azur, Var, Montauroux, Fondurane	N43.589, E6775	FR.MED
G.mar.75	ZFMKTW166	MG892188	France, Alpes-Côte d’Azur, Var, Montauroux, Fondurane	N43.589, E6775	FR.MED
G.mar.76	ZFMK2517199	MG931022	Spain, Pirineos, Le Grau	N42.412, E2.566	FR.PYRN
G.mar.77	ZFMK2517210	MG892161	France, Languedoc-Roussillon-Midi-Pyrénées, Ariege, Bas-Couserans	N42.997, E1.010	FR.PYRN
G.mar.78	ZFMK2517211	MG892162	France, Languedoc-Roussillon-Midi-Pyrénées, La Vallée de la Barousse	N43.017, E0.480	FR.PYRN
G.mar.79	ZFMK2517212	MG892163	France, Languedoc-Roussillon-Midi-Pyrénées, Le Canigou	N42.375, E2.456	FR.PYRN
G.mar.80	ZFMK19038	MG892135	Great Britain, England, Buckinghamshire	N51.750, W0.750	GB.EM
**Sequences from BOLD**					
G.mar.81	BOLDECHUB974		France, Haute Normandie, Seine-Maritime, Rouen, Foret verte	N49.500, E1.100	FR.ATLN
G.mar.82	BOLDECHUB975		France, Haute Normandie, Seine-Maritime, Rouen, Foret verte	N49.500, E1.100	FR.ATLN
G.mar.83	BOLDECHUB978		France, Haute Normandie, Seine-Maritime, Rouen, Foret verte	N49.500, E1.100	FR.ATLN
G.mar.84	BOLDECHUB979		France, Haute Normandie, Seine-Maritime, Rouen, Foret verte	N49.500, E1.100	FR.ATLN
G.mar.85	BOLDGENHP020		France, Haute Normandie, Seine-Maritime, Foret de Brotonne	N49.434, E0.714	FR.ATLN
G.mar.86	BOLDGENHP021		France, Haute Normandie, Seine-Maritime, Foret de Brotonne	N49.434, E0.714	FR.ATLN
G.mar.87	BOLDGENHP022		France, Haute Normandie, Seine-Maritime, Foret de Brotonne	N49.434, E0.714	FR.ATLN
G.mar.88	BOLDGENHP023		France, Haute Normandie, Seine-Maritime, Foret de Brotonne	N49.434, E0.714	FR.ATLN
G.mar.89	BOLDGENHP024		France, Haute Normandie, Seine-Maritime, Foret de Brotonne	N49.434, E0.714	FR.ATLN
G.mar.90	BOLDGENHP025		France, Haute Normandie, Seine-Maritime, Foret de Brotonne	N49.434, E0.714	FR.ATLN
G.mar.91	BOLDGENHP317		France, Haute Normandie, Seine-Maritime, Foret Henouville	N49.480, E0.954	FR.ATLN
**Sequences from GenBank**					
G.mar.92		FJ409909	Germany, Nordrhein-Westfalen, Bonn, Venusberg	N50.692, E7.100	DE.MGSW
G.mar.93		HM888107	Germany, Rheinland-Pfalz, Rheinbreitbach	N50.619, E7.254	DE.MGSW
G.mar.94		HM888108	Germany, Nordrhein-Westfalen, Bad Münstereifel	N50.560, E6.808	DE.MGSW
G.mar.95		HM888109	Germany, Rheinland-Pfalz, Rheinbreitbach	N50.619, E7.254	DE.MGSW
G.mar.96		HQ966136	Germany, Rheinland-Pfalz, Neustadt an der Weinstraße, Klausental	N49.392, E8.158	DE.SSL
G.mar.97		JQ350444	Spain, Navarra, Sierra De Urbasa	N42.830, W2.100	ES.CC
**Outgroup species/specimens**					
*Glomeris intermedia*					
G.int.1	see Spelda et al. 2011	HM888099	Germany, Rheinland-Pfalz, Neuwied		
G.int.2		HQ966138	Germany, Rheinland-Pfalz, Neustadt		
*Glomeris klugii*					
G.und.1	see Spelda et al. 2011	HM888106	Germany, Bayern, Lindau		
G.und.2		HQ966135	Germany, Bayern, Solnhofen		
*Glomeris connexa*					
G.con.1	see Spelda et al. 2011	HM888096	Germany, Bavaria, Andechs		
G.con.2		JN271879	Italy, Lombardia, Sondrio		
*Glomeris hexasticha*					
G.hex.1	ZFMK2542473	MG931024	Germany, Thüringen, Hermsdorf		
G.hex.2	ZFMK19526	MG931023	Germany, Bayern, Neumarkt		
*Glomeris maerens* species group					
G.mae.1	ZFMK2517198	MG892103	Spain, Valencia, Pego		
G.mae.2	ZFMK2517200	MG892104	Spain, Castellon, l’Alcora		
G.mae.3	ZFMK2517201	MG892105	Spain, Tarragona, Vandellos		
G.mae.4	ZFMK2517203	MG892106	Spain, Tarragona, Llaberia		
G.mae.5	ZFMK2517204	MG892107	Spain, Castellon, l’Alcora		
G.mae.6	ZFMK2517205	MG892108	Spain, Valencia, Pego		
G.mae.7	ZFMK2517206	MG892109	Spain, Tarragona, Reus, La Riba		
G.mae.8	ZFMK2517207	MG892110	Spain, Castellon, Atzeneta del Maestrat		
G.mae.9	ZFMK2517208	MG892111	Spain, Barcelona, Castellet, El Vendrell		
*Glomeris annulata*					
G.ann.1	ZFMKTW100	MG892190	France, Gard, Courry, 280-300 m		
G.ann.2	ZFMKTW101	MG892189	France, Gard, Courry, 280-300 m		
*Glomeris apuana*					
G.apu.1	ZFMKMYR752	KT188943	Italy, Liguria, Cinque Terre	see Wesener 2015	
G.apu.2	ZFMKMYR753	KT188944	Italy, Liguria, Cinque Terre		

**Figure 3.**
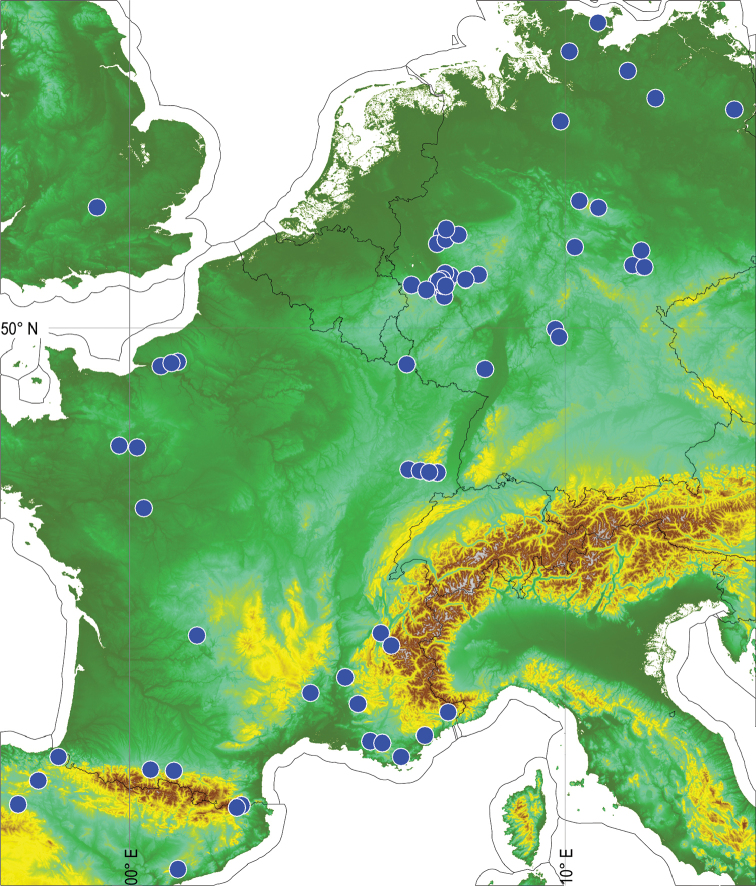
Distribution map of all successfully sequenced samples of *G.marginata* (blue dots). Base map: Shuttle Radar Topography Mission elevation data of the Deutsche Zentrum für Luft- und Raumfahrt (SRTM-3), accessed with GLOBALMAPPER v17.

The specimens of *G.marginata* were collected from a major part of the distribution region in NW Europe, covering the region from NE Spain to northern Germany (Figure 3). Material from the north-eastern part of the range (Denmark-Sweden-Norway) was not available. For the different analyses, two datasets were created, one which contained the 97 *G.marginata* sequences only, and a second one combining the *G.marginata* sequences with the 21 outgroup specimens.

### *DNA extraction, PCR, and sequencing*

From the analysed specimens, genomic mtDNA (the barcoding region of COI) was extracted from muscle tissue applying a standard extraction protocol (see e.g., Wesener et al. 2015) at the ZFMK. Also, the PCR and sequencing protocols were identical to those used in a previous work (Wesener et al. 2015). All specimens and the aliquots of the DNA extractions were deposited in the collection of the ZFMK. All new sequences (80 *G.marginata*, two *G.annulata*, and nine *G.maerens* sp. as *Glomeris* sp.) were deposited in GenBank (see Table 1 for accession numbers).

### Aligning and control

Sequences were aligned by hand in BIOEDIT (Hall 1999), version 7.2.5 (for final data set see Suppl. material S1). To rule out the accidental amplification of nuclear copies of the mitochondrial COI gene, the whole dataset was translated into amino acids following the ‘invertebrate’ code in MEGA 7 (Tamura et al. 2013); internal stop codons were absent in our dataset. There were in total 657 positions in the final dataset, gaps were absent. Voucher specimens and aliquots of the DNA extractions were stored in natural history collections and are available for each analysed sequence (see Table 1).

### Assignment to biogeographic regions

**Figure 4.**
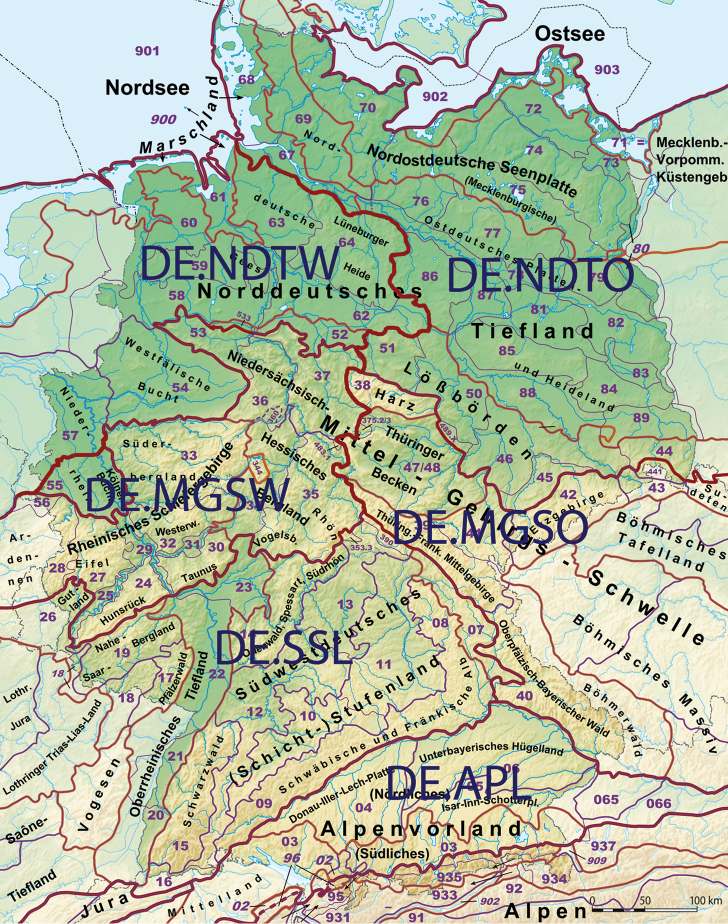
Modified biogeographic regions of Germany, based on Naturräumliche Großregionen of Germany, Meynen and Schmithüsen (1953–1962) and http://commons.wikimedia.org/wiki/File:Deutschland_Naturraeumliche_Grossregionen.png

**Figure 5.**
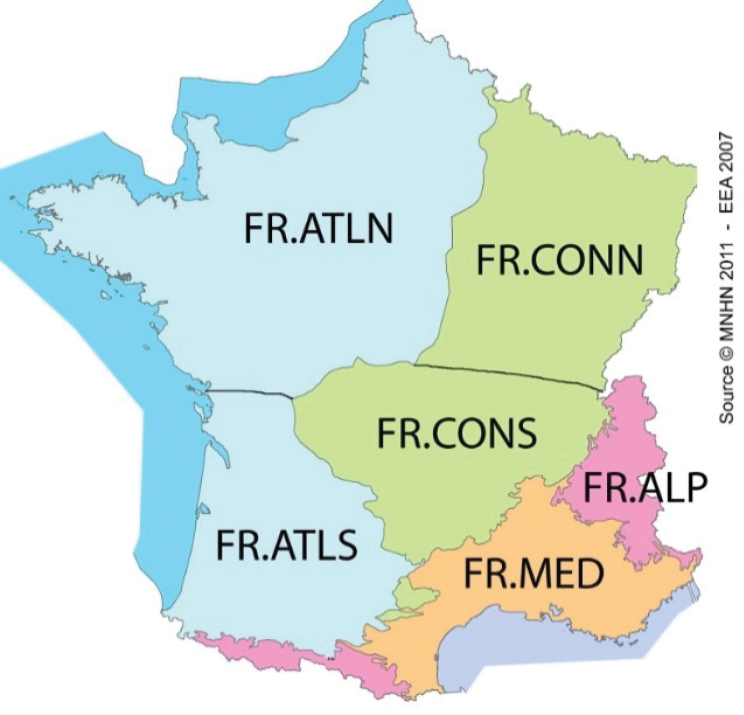
Modified biogeographic regions of France, based on http://inpn.mnhn.fr/programme/rapportage-directives-nature/presentation.

All specimens of *G.marginata* were assigned to a biogeographic region of the main sub-country level (bioregion) (see Table 1, column BioRegion and Table 2). The structuring of the specimens with their origin in Germany is based on the official map of natural regions of Germany, the “Großregionen”, 1^st^ level (Meynen and Schmithüsen 1953–1962, see also “Naturräumliche Großregionen Deutschlands” at http://de.wikipedia.org). Due to their disproportionately large size, the regions “Norddeutsches Tiefland“ and “Mittelgebirgsschwelle“ are additionally each divided into a western and eastern part according to Figure 4. The structuring of the specimens with their origin in France is based on the “régions biogéographiques pour l’évaluation de l’état de conservation en France” (see http://inpn.mnhn.fr/programme/rapportage-directives-nature/presentation). Additionally, the regions “France Atlantique“ and “France Continentale” – due to their size – are each divided into a northern and southern part as shown in Figure 5. The ecological region “France alpine” is geographically divided into France Alps and France Pyrenees. The single specimen from Great Britain is located in southern England. For Spain, we used the regions of southern Pyrenees and the Cantabrian Mountains. In total 14 biogeographic regions were assigned in four countries (see Table 2).

**Table 2.** Biogeographic regions (bioregions) and their code.

Region code	Region
**Germany**	
DE.NDTW	“Norddeutsches Tiefland” western part, Norddeutsche Geest west of river Elbe
DE.NDTO	“Norddeutsches Tiefland” eastern part, east of river Elbe
DE.MGSW	“Mittelgebirgsschwelle”, western part, Niedersächsisch-Hessisches Bergland, Rheinisches Schiefergebirge, Kölner Bucht
DE.MGSO	“Mittelgebirgsschwelle”, eastern part, Harz, Thüringer Becken, Östliche Mittelgebirgsschwelle
DE.SSL	“Schichtstufenland” on both sides of the Oberrheingraben
**France**	
FR.CONN	France Continentale, northern part
FR.CONS	France Continentale, southern part
FR.MED	France Méditerranéenne
FR.ATLN	France Atlantique, north of La Rochelle
FR.ALP	Alps of France
FR.PYRN	Pyrenees of France
**Spain**	
ES.PYRS	Pyrenees of Spain
ES.CC	Cordillera Cantábrica (Navarre, Sierra de Urbasa)
**Great Britain**	
GB.EM	Middle England

### Phylogenetic and distance analysis

Analyses were conducted in MEGA 7 (Kumar et al. 2015). The uncorrected pairwise distances (p-distances) were calculated with all codon positions included. Ambiguous positions were removed for each sequence pair. The distance matrix was exported to MICROSOFT EXCEL for further calculations of minimum interspecific and maximum intraspecific distances (see Suppl. material S2).

A model test, as implemented in MEGA 7, was performed to find the best fitting maximum likelihood substitution model for the complete sequence set. The model with the lowest AICc value (Akaike Information Criterion, corrected) are considered to describe the best substitution pattern. Codon positions included were 1^st^ + 2^nd^ + 3^rd^. The model test selected the General Time Reversible model (Tavaré 1986) with gamma distribution and invariant sites (GTR+G+I) as the best fitting model (AIC: 7988, lnL: -3750).

The evolutionary history was inferred by using the maximum likelihood method based on the selected GTR+G+I model. Initial tree(s) for the heuristic search were obtained automatically by applying NJ/BioNJ algorithms to a matrix of pairwise distances estimated using the Maximum Composite Likelihood (MCL) approach, and then selecting the topology with superior log likelihood value. The discrete gamma distribution was used with five categories to model evolutionary rate differences among sites. The analysis involved the complete sequence set (*G.marginata* + outgroup species). Codon positions included were “1st+2nd+3rd” (Missing Data: partial deletion). The bootstrap consensus tree inferred from 1,000 replicates (Felsenstein 1985) is taken to represent the evolutionary history of the analysed taxa. Trees were built with FIGTREE 1.4.2 and drawn to scale, with branch lengths measured in the number of substitutions per site.

## Spatial relationship

Besides the genetic p-distances (see above) for all *G.marginata* specimen pairs (4656 pairs) the geographical distances were calculated based on the more exact method of calculation, the Euclidean geometry:


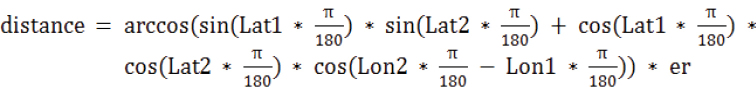


The earth’s radius (= er) in central Europe is 6,367 km. Lat1 and Lon1 are the latitude and longitude of the location of specimen 1, Lat2 and Lon2 those of specimen 2. For the full dataset see Suppl. material S3. A chart was plotted to show the relationship between the genetic and geographical distance.

### Haplotype analysis

A haplotype analysis was conducted with DNASP (Librado and Rozas 2009) by assigning the genetic code to “mtDNA *Drosophila*” for invertebrates. The *G.marginata* sequences were grouped to haplotypes (DNASP / Generate / HaploType Data File, excluding sites with missing data). The haplotypes were marked by geography.

In a second run the sequences were grouped again by considering only non-synonymous changes. In this second step all synonymous changes were discarded. For this an interim sequence set with only non-synonymous changes was created (DNASP / Generate / Polymorphic Data File / “only Non-synonymous”) and afterwards the Haplotype file was built. Because of the unequal sampling with a bias to the German fauna within the GBOL-project, no comparative population analysis was possible.

The previous first haplotype data file was used as a basis for a TCS Networks analysis (Clement et al. 2002). A TCS-network was created with the software POPART (Leigh 2015). For this a frequency matrix of haplotypes to bioregions was created in MICROSOFT EXCEL and according the software manual transformed to the POPART-nexus format (see Suppl. material S4).

### Haplotype richness estimation

The potential number of haplotypes for the complete distribution area was estimated with ESTIMATES 9.1.0 (Colwell 2013). For this, the CHAO1-estimator (Chao 1984) based on the haplotype distribution (instead of a species distribution) was calculated (for the underlying data file see Suppl. Material S5). Together with the ACE-index the CHAO1-estimator is the main estimator for individually based abundance data (Gotelli and Colwell 2010). It is based on the number of all OTUs (operational taxonomic units, in this study the haplotypes) with one sequence in relation to the number of all OTUs with two sequences. With 10,000 randomized runs the haplotype accumulation curve (rarefaction curve) and the 95 % lower and upper boundaries of confidence intervals were calculated and additionally also their extrapolation curves (formulas in detail see Colwell et al. 2012).

## Results

### Phylogenetic relationship of *G.marginata* with similar species

**Figure 6.**
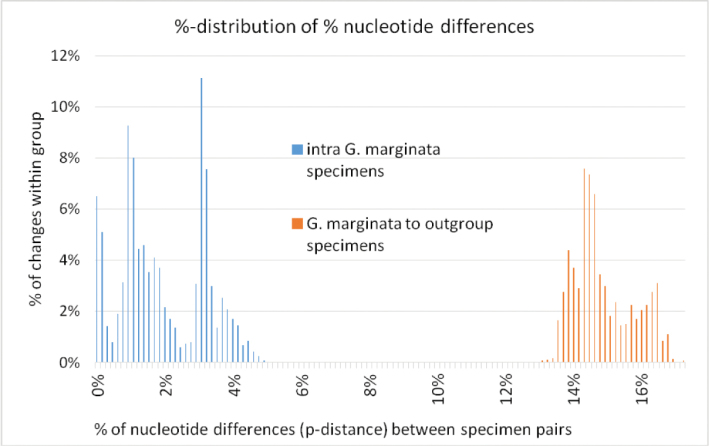
Distribution of nucleotide differences in % between specimen pairs of *Glomerismarginata* and to outgroup specimens.

**Figure 7.**
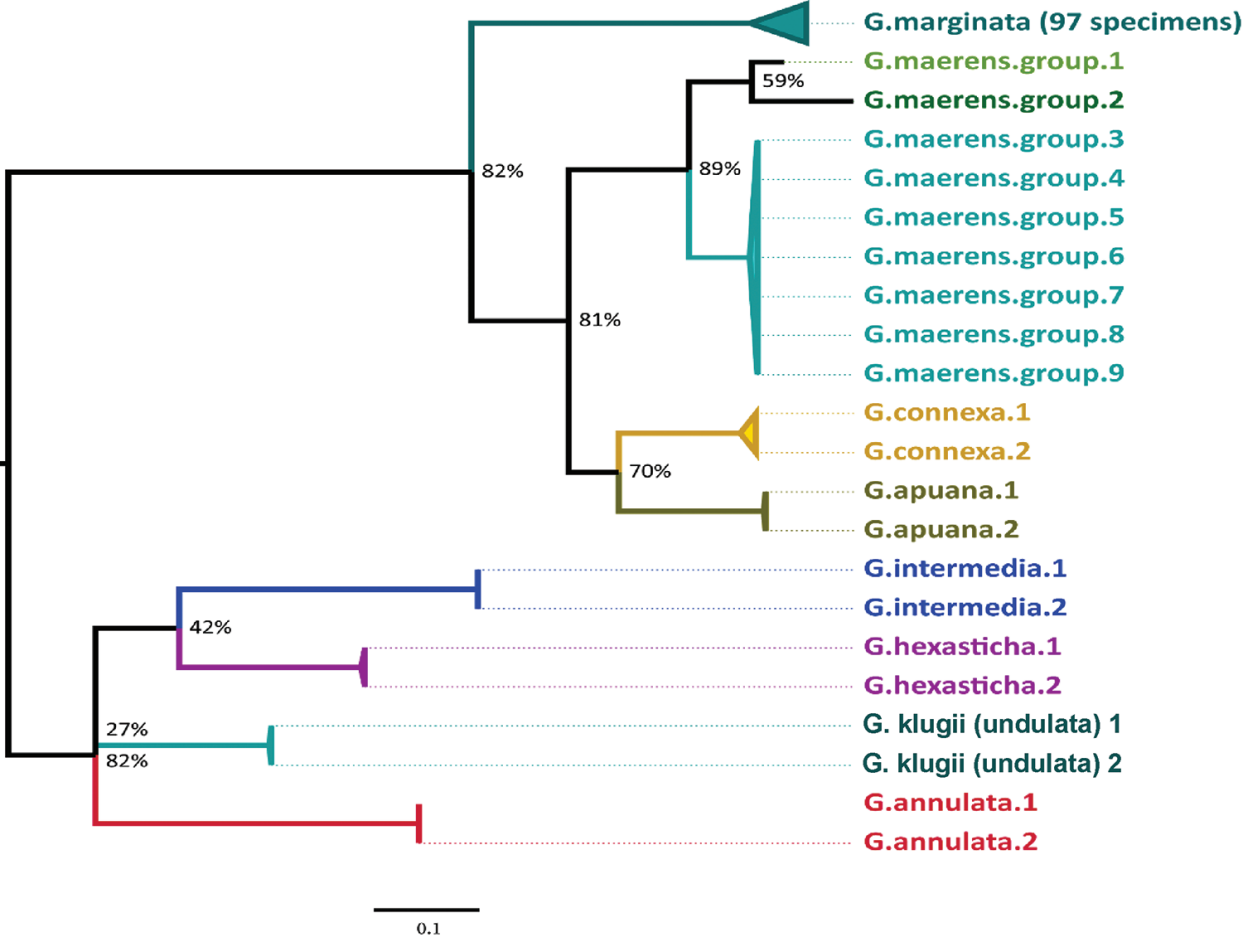
Molecular phylogenetic analysis of *Glomeris* species by the maximum likelihood method. Midpoint rooted. Bootstrap values in % at nodes. All collapsed nodes have a bootstrap value of 100%. Scale bar: 10 % genetic ML distance.

The minimum interspecific distance of *G.marginata* to other *Glomeris* species ranges from 12.9–15.9 % (see Table 3). There is a clear barcoding gap between the maximum intraspecific distance (5.0 %) and the minimum interspecific distance (12.9 %) (see also Figure 6). *Glomerisconnexa* and the *G.maerens* species-group are closest to *G.marginata*. The separation of the outgroup species to *G.marginata* is clearly visible in the graphical mapping of the phylogenetic analyses (see Figure 7). The *G.marginata* specimens, together with *G.connexa*, *G.apuana*, and the *G.maerens*-group, form a distinct clade separate from the other species. The other four species (*G.hexasticha*, *G.klugii*/*undulata*, *G.intermedia*, and *G.annulata*) form a single clade. Statistical support for both clades is rather low, not exceeding 82 %.

**Table 3.** Minimum p-distance of *G.marginata* to other species.

Species	Min. p-distance to *G.marginata*
* Glomeris connexa *	12.9 %
*Glomerismaerens*-group	13.1 %
*Glomerisklugii*/undulata	13.4 %
* Glomeris apuana *	14.2 %
* Glomeris intermedia *	14.8 %
* Glomeris hexasticha *	14.9 %
* Glomeris annulata *	15.9 %

The specimens of the *G.maerens* species-group cluster together with a minimum interspecific distance (10.5 %) to the other species, but the *G.maerens* specimens fall into three clades with a maximum intraspecific distance of up to 9.1 % (see Figure 7).

### Intraspecific variation of *G.marginata*

All 97 specimens of *G.marginata* form a well-supported clade (bootstrap value 100 %, not shown in Figure 7). The 97 specimens of *G.marginata* have a maximum intraspecific distance of 5.0 %. The intraspecific distance chart (Figure 6, blue bars) shows three peaks (at: 0 %, 0.9 % and 3.0 %) within the p-distances of the *G.marginata* specimens; within the range every p-distance value is present. There is no gap in the distribution of the p-distance values.

### Geographical relationship of *G.marginata* specimens

The specimens from northern Germany and eastern France show the lowest genetic distance (≈ 1 %) to the rest of all samples. The specimens from western and southern France show the highest median distance (≈ 3–4 %) to those of other populations (see Table 4 and Suppl. Material S2).

**Table 4.** The 10 specimens with smallest and greatest median p-distance to the rest of samples.

		p-Distance		
**SpecimenID**	**BioRegion**	**Median**	**Max**	**Mean**
G.mar.40	DE.NDTO	0.6 %	3.5 %	1.2 %
G.mar.17	DE.MGSW	0.9 %	4.0 %	1.3 %
G.mar.58	FR.CON	0.9 %	3.8 %	1.4 %
G.mar.59	FR.CON	0.9 %	3.8 %	1.4 %
G.mar.61	FR.CON	0.9 %	3.8 %	1.4 %
G.mar.95	DE.MGSW	0.9 %	3.8 %	1.4 %
G.mar.04	DE.MGSO	1.1 %	3.8 %	1.4 %
G.mar.05	DE.MGSO	1.1 %	3.8 %	1.4 %
G.mar.06	DE.MGSO	1.1 %	3.8 %	1.4 %
G.mar.07	DE.MGSO	1.1 %	3.8 %	1.4 %
…	…	…	…	…
G.mar.85	FR.ATLN	3.2 %	4.9 %	2.8 %
G.mar.86	FR.ATLN	3.2 %	4.9 %	2.8 %
G.mar.68	FR.MED	3.3 %	4.7 %	3.4 %
G.mar.65	FR.MED	3.5 %	4.6 %	3.4 %
G.mar.66	FR.MED	3.5 %	4.6 %	3.4 %
G.mar.67	FR.MED	3.5 %	4.6 %	3.4 %
G.mar.79	FR.PYRN	3.8 %	4.9 %	3.7 %
G.mar.77	FR.PYRN	3.8 %	4.6 %	3.8 %
G.mar.76	FR.PYRN	4.0 %	5.0 %	3.9 %
G.mar.71	FR.MED	4.0 %	5.0 %	3.9 %

The maximum and the mean p-distance of *G.marginata* within the north-eastern part of the distribution (≈ 4 % or ≈ 1 %, respectively) is lower than in the south-western part (≈ 5 % or ≈ 3–4 %, respectively). Specimens from Mediterranean France group most distantly from the rest, with a maximum p-distance of 5.0 %.

**Figure 8.**
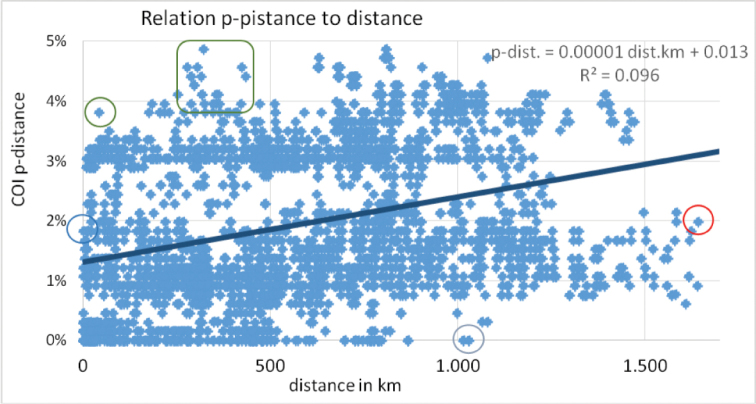
Mapped genetic p-distance to geographical distance of all analysed specimen-pairs (4,656) of *Glomerismarginata*. Solid line: linear trend line with linear function and coefficient of determination R². Circles: see text below and Table 5.

The plot of the genetic p-distance to the geographical distances of all samples (4,656 possible pairs) shows no distinct relationship between both values (see Figure 8). There is a small and negligible trend of +0.00001 % p-distance/km-distance. The coefficient of determination R² with ≈ 0.1 is extremely low. For example, two specimens collected only 43 km apart (77 to 78, see Figure 8: green circle and Table 5) show a genetic p-distance of 3.8 %, while contrarily two specimens with a geographical distance of more than 1,000 km (43 to 54, see Figure 8, grey circle and Table 5) belong to an identical haplotype (0 % p-distance). The geographically most distant analysed specimens (41 to 49, Figure 8: red circle) show a p-distance of 2.1 %.

**Table 5.** Examples of specimen pairs with small and great ratio of p-distance (p-dist.) to geographical distance (geo-dist in km). Green marked: specimen pairs with exceptionally high p-dist. but low geo-dist. (representative for dots of upper-left side of Figure 8: green box). Light-blue marked: specimens of the same location with the highest p-dist (Figure 8: blue circle). Orange marked: specimen pair with exceptionally low p-dist. but high geo-dist. (representative for dots of lower-right side of Figure 8: red circle). Grey-blue marked: most distant specimen pair with identical haplotype (Figure 8: grey circle).

SpecimenID	SpecimenID	geo-dist	p-dist	p-dist./geo-dist.
G.mar.71 (FR.MED)	G.mar.79 (FR.PYRN)	322	4.9 %	0.000151
G.mar.77 (FR.PYRN)	G.mar.78 (FR.PYRN)	43	3.8 %	0.000883
G.mar.26 (DE.MGSW)	G.mar.93 (DE.MGSW)	9	3.0 %	0.003204
G.mar.26 (DE.MGSW)	G.mar.36 (DE.MGSW)	8	2.9 %	0.003486
G.mar.30 (DE.MGSW)	G.mar.31 (DE.MGSW)	0	1,8 %	–
…	…	…	…	…
G.mar.57 (FR.ATLN)	G.mar.74 (FR.MED)	647	0.2 %	0.000002
G.mar.01 (DE.MGSO)	G.mar.54 (FR.ALP)	820	0.2 %	0.000002
G.mar.44 (DE.NDTO)	G.mar.84 (FR.ATLN)	868	0.0 %	–
G.mar.43 (DE.NDTO)	G.mar.54 (FR.ALP)	1031	0.0 %	–
G.mar.40 (DE.NDTO)	G.mar.52 (ES.PYRS)	1610	0.6 %	0.000004

### Haplotypes/regions

Within the 657 sites of the 97 sequences of *G.marginata*, 74 were polymorphic which resulted from a total number of 81 mutations. The total number of synonymous changes is 71 and the total number of replacement changes is six. In the haplotype analysis, within the 97 samples, 47 haplotypes were detected, with 79 polymorphic sites. Haplotype diversity is 0.93, nucleotide diversity Pi is 0.017.

38 haplotypes (81 % of all haplotypes) consist of only one specimen (^ = 38 specimens ≙ 39 % of all specimens) and 42 haplotypes (89 % of all haplotypes) represents only specimens from one bioregion (^ = 48 specimens ^ = 49 % of all specimens). Nine haplotypes are represented in our dataset with two or more specimens (^ = 59 specimens ^ = 61 % of all specimens).

**Figure 9.**
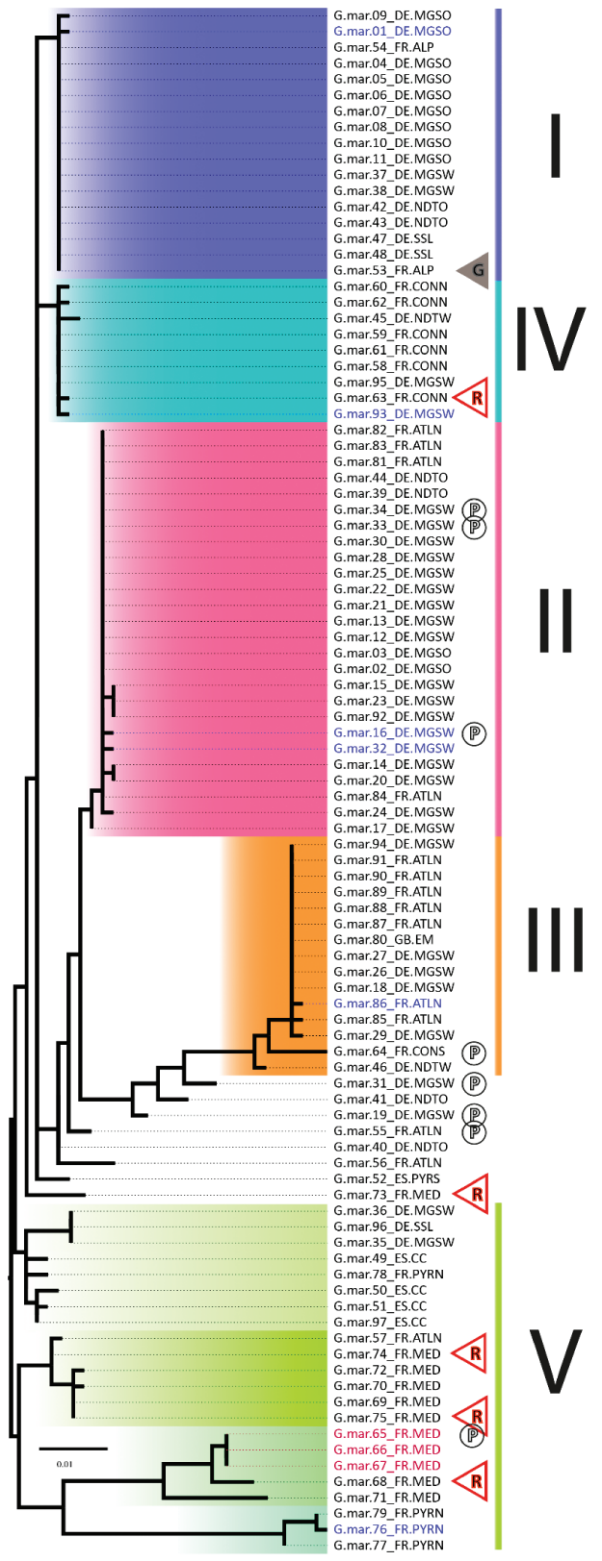
Maximum likelihood tree based on the mtDNA COI gene of 97 *Glomerismarginata*. Midpoint rooted. Roman numerals: Haplotype lineages I–V. Colour morphs of *G.marginata*: Common black = none; G = grey; R = red border; P = perplexa-markings. Scale bar = 1 % genetic ML distance.

**Figure 10.**
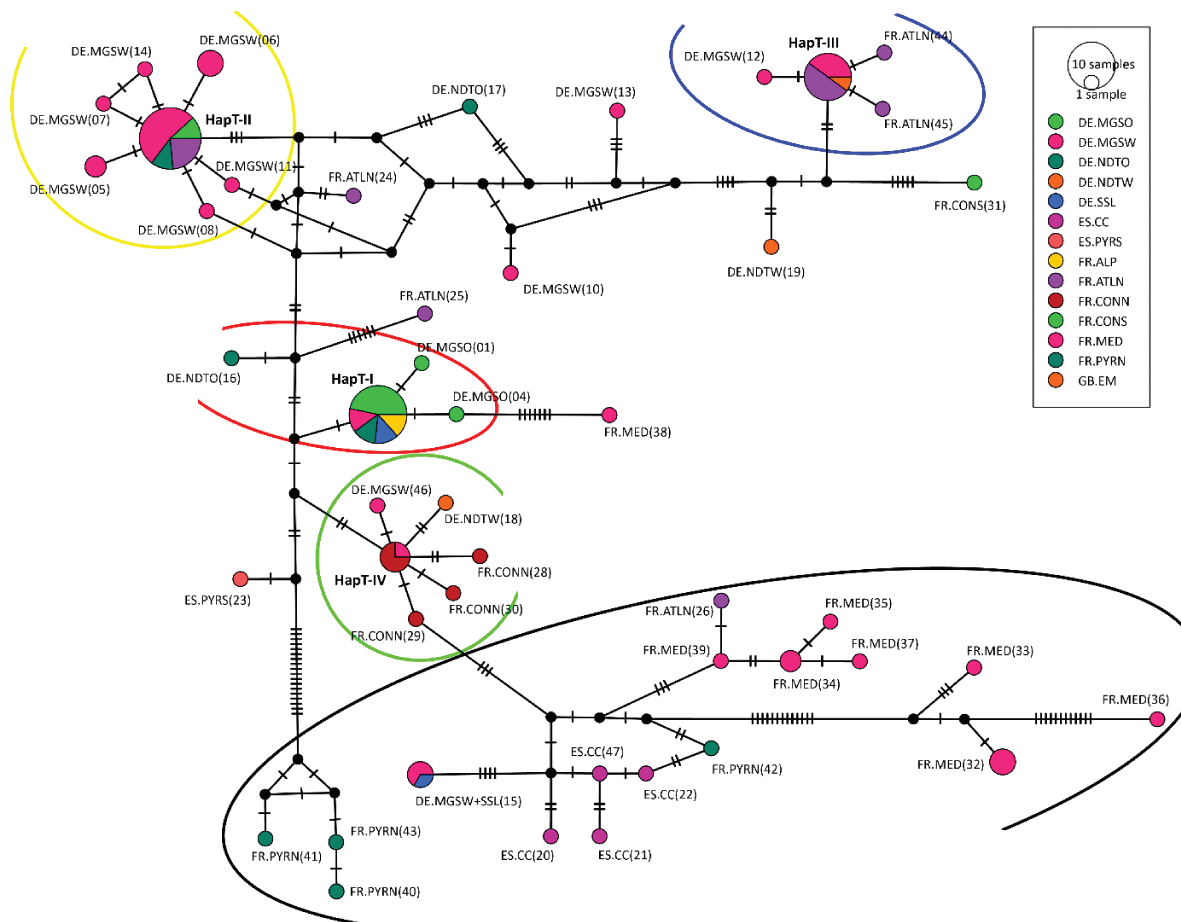
TCS-Network of haplotypes of *Glomerismarginata* with distribution region. Numbers behind region = consecutive haplotype number of DNASP-output. Haplotype accumulations: Red oval = Haplotype lineage I; Yellow oval = Haplotype lineage II; Blue oval = Haplotype lineage III; Green circle = Haplotype lineage IV; Black oval = Haplotype lineage V. Dashes on node connecting lines are representing single nucleotide mutations.

The dataset was divided into five major haplotype lineages (see Figure 9 and partially Table 6). The major haplotype lineage V is basal to all other and shows a higher internal genetic variability (to their member subgroups and specimens: Ø 2.4 %) than the other haplotype lineages of *G.marginata*. Haplotype lineage V consists of several loosely connected subgroups, mainly from the French Mediterranean, the French Pyrenees and Spanish Cantabria (FR.MED, FR.PYR and ES.CC) (see Figure 9, Figure 10, black circled). This basal group is connected to the bioregion DE.MGSW via specimens 35 and 36 (Figure 8, Table 1). The area occupied by lineage V excludes all other major haplotype lineages, which do not extend to the two South French regions (FR.MED and FR.PYR), or to the more western Spanish Cantabrian Mountains (ES.CC).

**Table 6.** Number of samples and bioregions (BioR) to major haplotypes (mHapT) and lineages.

Number of lineages in Figure 9	Number of Samples in mHapT	Number of BioR/mHapT BioR/lineage	Covered BioR	Number of Samples/lineages
**I**	**15**	**5**	**DE.MGSW – DE.MGSO – DE.NDTO DE.SSL – FR.ALP**	**17**
**II**	**17**	**4**	**DE.MGSW – DE.MGSO – DE.NDTO FR.ATLN**	**26**
**III**	**10**	**3/5**	**DE.MGSW – FR.ATLN – GB.EM** *DE.NDTW – FR.CONS*	**15**
IV	4	2/3	DE.MGSW – FR.CONN – *DE.NDTW*	9

The other four haplotype lineages I–IV show a wider area of distribution, but genetically less diversity. Major haplotype lineages I and IV are closely related (see Figure 9). Together this joint lineage (I+IV) covers almost the complete northern distribution range of *G.marginata* (seven bioregions: DE.MGSO, DE.MGSW, DE.NDTO, DE.NDTW, DE.SSL, FR.CONN, and FR.ALP see Table 6).

Haplotype lineage I occurs in an area reaching from the French Alps to NE Europe, with the main haplotype diversity in the German “Mittelgebirgsschwelle“, eastern part (DE.MGSO). Haplotype lineage II shows a central distribution with a high proportion of specimens in the German “Mittelgebirgsschwelle“, western part (DE.MGSW). Lineage II has the greatest distribution area and includes several subordinated haplotypes in the region DE.MGSW. Haplotype lineage III occurs in NW Europe with the most specimens in the France Atlantique, northern part (FR.ATLN). Additionally, the specimen from Great Britain (GB.EM) belongs to this group and has even the same haplotype as the majority specimens of this lineage. Haplotype lineage IV has a more narrow distribution range, with its main samples in France Continentale, northern part (FR.CONN). None of those four lineages are found in southern France or northern Spain (the distribution area of lineage V), but the distribution areas of the lineages I–IV overlap in DE.MGSW.

Haplotype lineages I–III and partially lineage IV are especially poor in haplotypes. Four haplotypes, one in each lineage (see Table 6), are especially rich in specimens, 17, 15, 10, and 4, respectively, together representing 47 % (46 specimens) of all analysed *G.marginata*. Additional haplotypes can be added to those four main haplotypes, differing only by a few basepairs. 65 specimens can therefore be grouped into these haplotype lineages (I–IV in Table 6 and Figure 8, ^ = 67 % of all specimens).

Every well-sampled bioregion has many haplotypes. The haplotype/specimen-rate is always higher than 0.3 (see Table 7). The less sampled a region is, the higher the haplotype/samples rate is. At the French Pyrenees and the Spanish Cantabrian Mountains, every sample of *G.marginata* represents a different haplotype. The three especially well-represented major haplotypes of lineages I-III were collected in 5, 4 or 3 different bioregions (see Table 6). These three haplotypes/lineages each cover a large geographical range, with all three overlapping centrally in the bioregion DE.MGSW, our best-sampled region.

The haplotype lineage III mainly connects the northern French bioregion (FR.ATLN) with central Germany (DE.MGSW). One direct connection exists between the southern French/Spanish (FR.MED, FR.PYR and ES.CC) and the northern French populations (specimen 57, FR.ATLN, Table 1).

**Table 7.** Rates of haplotypes (HapT) and haplogroups (HapG) per samples in major sampled bioregions (BioR).

BioRegion	Samples in BioR	HapT in BioR	HapT/Samples	Mean p-distance	HapG in BioR	HapG/Samples
Total	97	47	*0.5*	1.9 %	8	0.1
DE.MGSW	31	15	*0.5*	1.4 %	*4*	*0.1*
FR.ATLN	14	7	*0.5*	1.9 %	*2*	*0.1*
DE.MGSO	11	4	*0.4*	0.4 %	*2*	*0.2*
FR.MED	11	8	*0.7*	2.2 %	*2*	*0.2*
DE.NDTO	6	4	*0.7*	0.8 %	*1*	*0.2*
FR.CONN	6	4	*0.7*	0.2 %	*1*	*0.2*
ES.CC	4	4	*1.0*	0.6 %	*1*	*0.3*
FR.PYRN	4	4	*1.0*	2.1 %	2	0.5
N-Europe	77	30	*0.4*	1.8 %	6	*0.1*
S-Europe	20	17	*0.9*	2.5 %	3	*0.2*

### Haplotype network of *G.marginata*

Based on the 47 haplotypes the TCS analysis shows a complex net of different possible evolutionary pathways between the haplotypes (see Figure 10). The clustering of the main four haplotypes (four largest filled circles in Figure 10) is similar to our phylogenetic tree (Figure 9), with adjacent and closely related haplotypes forming distinct lineages (coloured oval lines in Figure 10). The haplotypes of the southern Mediterranean France and southern Spain are building a complex, highly disjunctive net (black oval line in Figure 10).

### Haplotype number estimation

**Figure 11.**
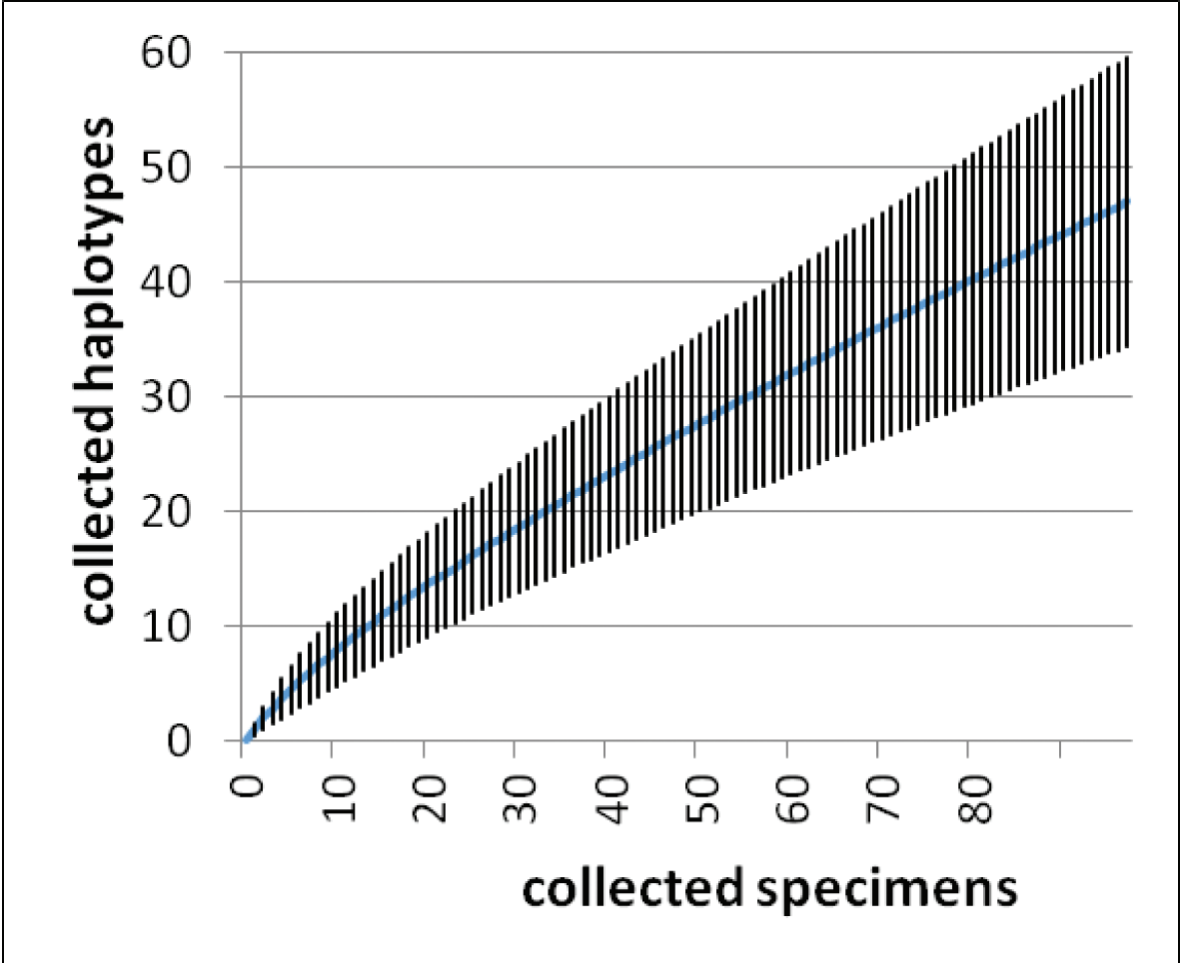
Individual based rarefaction curve calculated with ESTIMATES and with 10,000 replicates (simulated collections) of the COI sequences of *Glomerismarginata*. Vertical lines indicating 95% lower and upper boundary.

**Figure 12.**
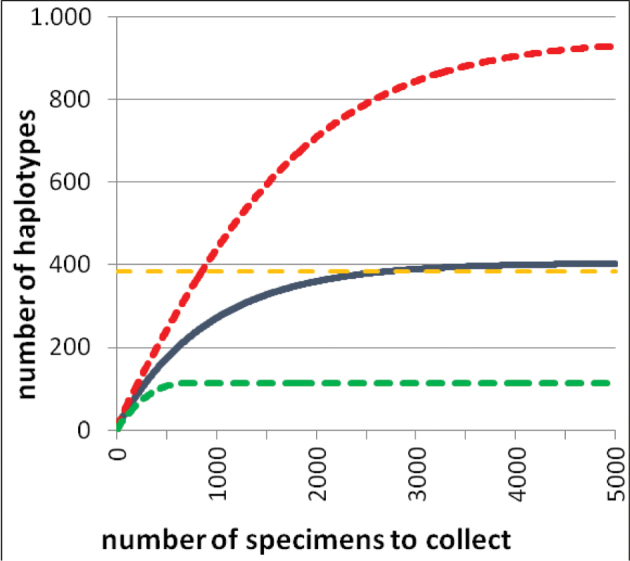
Extrapolation of rarefaction curves with ESTIMATES of the COI sequences of *Glomerismarginata*. Blue line = estimation with premise of mean number (404 haplotypes); Horizontal yellow line = 95% satisfaction of mean number (384 haplotypes); Green and red line = curve at the 95% upper and lower boundary.

The rarefaction curve shows no saturation for the number of haplotypes (see Figure 11, 12). The estimation of CHAO1 shows that there could be overall 404 haplotypes in *G.marginata* (95 % confidence interval: 140–1,426 haplotypes). By extrapolation with rarefaction curves (Colwell et al. 2012) we estimate that a mean of 6,612 samples would be needed to be analysed to find all potential 404 different haplotypes. To reach the 95 % lower boundary (140 haplotypes) at least an additional 274 specimens need to be included.

### Colour morphs of *G.marginata*

The dataset contains one specimen of the grey colour morph, eight with the “perplexa” pattern and four with red margins. Those 13 distinctly coloured specimens are marked in our specimen tree (see Figure 9 with symbols “G”, “P”, and “R”). The grey specimen belongs to the major haplotype of the lineage I. The specimens with the red margin are scattered in the tree and therefore do not cluster together. They are mainly found in Mediterranean France, therefore placed mainly in the lumping group V, but one specimen groups with lineage IV (Figure 9). The “perplexa” form is even more scattered over the tree, occurring in several bioregions.

## Discussion

### *Glomerisannulata*, *G.apuana*, and *G.maerens*

The three local endemic species, despite some similarities in the coloration (Figures 2D–G), are genetically clearly distinct from *G.marginata*, separated by p-distances of more than 13 %.

Further studies should investigate the *G.maerens*-group in northern Spain. All three species (*G.maerens*, *G.lugubris* Attems, 1927, and *G.obsoleta* Attems, 1952) of the group were described by Attems from Spain (*G.maerens*: Tarragona and Lérida; *G.lugubris*: Cádiz; *G.obsoleta*: Barcelona) and show a similar obscure black-brown colouration (see examples in Figure 2E–G). Due to their geographically close type locations and quite similar colour, as well as thoracic shield striation pattern (both with two main striae) *G.maerens* and *G.obsoleta* may be synonyms. Therefore, the examined specimens could not be assigned to either species. However, our analysis recovers a considerable variation inside the species-group, with p-distances of 7.5–9.1 % which hints at the existence of several independent species in the *G.maerens* complex.

### Monophyly of *G.marginata*

*Glomerismarginata* is genetically distant but related to *G.connexa*, with a p-distance of 12.9 %. Based on the COI-data, the *G.maerens* species group is more closely related to *G.connexa*/*G.apuana* than to *G.marginata*. The genetic distance of *G.marginata* to the other tested species (*G.klugii/undulata*, *G.intermedia*, *G.hexasticha*, and *G.annulata*) is, with a p-distance up to 15.9 %, even more pronounced.

In comparison to vertebrate species (e.g., fishes: 0.32 %, Keskin and Atar 2013 or rodents: 2.1 %, Li et al. 2015) a maximum intraspecific variation of a p-distance of 5 % is rather high. However, such an intraspecific variation of 5 % was also found in another widespread central European *Glomeris*, *G.klugii/undulata* (Wesener and Conrad 2016). A minimum p-distance of 12.9 % of *G.marginata* to the most closely related species (a factor of 2.6 to the maximum intraspecific p-distance), shows a clear barcoding gap to the nearest congener, *G.connexa*.

The known colour morphs of *G.marginata* do not represent single lineages or even subspecies. The conspicuously red borders in specimens from southern France (Figures 1D, E) are present in several lineages and sub-lineages (Figure 9, marked with R). The same applies to the perplexa-form (Figures 1A, B, 9, marked with P). The grey form is even a member of the main haplotype of the eastern lineage I (Figure 9, marked with G). Unfortunately, specimens of the brown form of northern Germany could not yet be sequenced, but they appear always syntopically with specimens of the black form (Figure 2A). Therefore, any relevant divergence from those haplotypes cannot be expected.

The COI-gene is clearly working as a barcoding gene to identify and discriminate *G.marginata* specimens from the other *Glomeris* species.

### Geographical relationship of *G.marginata* specimens

Syntopical specimens as well as specimens with a maximum geographical distance of 1,701 km (Germany, Brandenburg to Spain, La Rioja) were analysed. There is no obvious relationship between geographical and genetic distance. There are specimen pairs of the same haplotype (p-distance = 0) which were collected more than 1,000 km apart. This distance of 1,000 km seems to be the maximum distance *G.marginata* could spread without experiencing genetic changes. Specimen pairs with a geographical distance larger than 1,000 km experienced at least a few mutations in the COI gene, with a minimum p-distance of ≈ 0.8 % in our dataset (see Figure 7).

On the other hand, local specimens can show high genetic variation. Even from nearby locations specimen pairs show a p-distance as high as 3 %. Such a mutation rate is unlikely to have happened locally, but is more likely the result of a different geographical origin of the source populations. As such large genetic distances between different populations of *G.marginata* are common, a human-influenced dispersal seems not to be the reason behind the regular high COI-variance.

### Haplotype regions, origin and potential migration patterns

The haplotype analysis shows five main haplotype lineages in *G.marginata* (Figure 9). Four of those (I–IV) show a wide distribution in northern Europe, one (lineage V) is restricted to southern Europe.

The haplotype lineage V is highly genetically variable, therefore a combination into a single group is not justified. Four rather distinct lineages not forming a monophylum could be seen in Figure 9 (coloured in different shades of green). Additionally, a block with unrelated singular haplotypes (see Figure 9 between lineage III and V) could be assigned to this fifth major haplotype lineage. Most of the specimens of these unrelated singular haplotypes are coming from the Mediterranean. These unrelated haplotypes are linked to the region DE.MGSW (specimens 19 and 31; Figure 8).

The examined northern European regions are mainly inhabited by specimens of the haplotype lineages I–IV, showing a low variance in their p-distance to one another (see Table 7). The specimen pairs within the whole North European area have a mean p-distance of 1.8 %. In contrast the French Mediterranean and French Pyrenees specimens of *G.marginata* show a higher p-distance (FR.MED: 2.2 % and FR.PYRN: 2.1 %). The specimen pairs of *G.marginata* within the geographically smaller South European bioregions (FR.MED, FR.PYRN, ES.PYRS, and ES.CC) have a mean p-distance of 2.5 %, higher than those observed in the entire North of Europe (1.8 %). With further sampling in southern Europe and collecting of similar haplotypes those values might decrease, however, further sampling will also reveal new haplotypes (see Figure 11). A saturation of the number of haplotypes is not detectable (see Figures 11, 12).

With the before mentioned mean p-distance of 2.5 %, the small south European area of bioregions contains a much higher genetic diversity in *G.marginata* than the much larger northern Europe. To develop such a higher genetic diversity, the south European populations of *G.marginata* must be older than the northern European populations. Northern Europe must have been colonized by *G.marginata* more recently. The main dispersal into those northern areas could only have been started after the last glaciation retreated during the early Holocene starting around 11,000 years ago (Roberts 2014).

Our data does not reveal how far north the distribution of *G.marginata* reached and how high any genetic diversity of the species was before the ice age. However, the south European mixed populations could be regarded as a remnant of old haplotype lineages of *G.marginata*, which are not any more present in the north European populations.

The geographical coverage of our analysed specimens is biased towards western Germany (MGSW, see Figure 3). For the colonization of northern Europe there are two possible scenarios. The new dispersal could have started from the south, or the dispersal could have started from a glacial refugium in northern Europe. The two scenarios are, however, not mutually exclusive and could have been concurrent. From a genetic point of view the northern populations differ from the southern populations. There are only a few and weak links between north and south. Therefore, a single or main colonization from the south to the north is not plausible.

Contrarily, all main haplotype lineages I–IV, which are exclusively found in northern Europe are linked to the bioregion DE.MGSW (Figure 3). The main redistribution over northern Europe could have been started from central Germany, which shows high haplotype diversity in *G.marginata*. From the bioregion DE.MGSW four major migrations could have led to the current distribution of the main haplotype lineages I–IV. Haplotype lineage I might have spread mainly to the north-east, haplotype lineage III to the North-West and haplotype lineage II only westwards. Haplotype-lineage IV spread to the bioregion FR.CONN. The colonisations by the haplotype lineages were probably independent.

### Haplotype number estimation

With this work, for the first time, a survey of almost 100 barcodes is presented for a diplopod species. On average, every haplotype in our study is based on two specimens (97 specimens / 47 haplotypes). In reality, the majority of haplotypes (38 haplotypes ^ = 81 %) are represented by only one specimen. The haplotype number estimation has shown that these 97 successfully sequenced specimens are just providing an overview of the real haplotype diversity in *G.marginata*. With the current data we are still far away from a complete collection of all haplotypes of the species. Many more specimens need to be collected to reach at least the lower estimated boundary of 140 haplotypes.

In general, this also means that haplotype analysis should not be based on few specimens and not only on specimens of a certain region, but always from specimens covering the whole distribution area of a species (Elias et al. 2007, Bergsten et al. 2012, Jordal and Kambestadt 2014). With the current data we should have a good base to cover the whole range of haplotypes. Further new haplotypes should mainly cluster within the current main lineages I to IV or should end up within the haplotype complex V with its four subgroups.

Many new haplotypes would simply represent the missing mutation steps present in the TCS-network of Figure 10 by dashes between the nodes. Probably most of the haplotypes representing end nodes in the current TCS-network are not representing the real end nodes of the mutation chains.

### Nomenclatorial acts

In the year 1789 the species with the common name Cloporte bordé (bordered woodlouse) was first described by the French naturalist Charles Joseph de Villers (1724–1810) as *Oniscusmarginatus*. He used few, but descriptive words: “niger, segmentis corporis luteo marginatis” [black, segments of the body with yellow margin].

Within a few years the species has been named and described four times again (see below). Thirteen years after the description the French zoologist Pierre André Latreille (1762–1833) placed the species in his new genus *Glomeris* Latreille, 1802. Almost one hundred years later several subspecies or variations were added by Verhoeff, Latzel, and Attems. Those taxa represent different versions of the pale form which was first named *G.perplexa* by Latzel (1895), all now regarded as synonyms of the nominate species.

We do not recognize any subspecies of *G.marginata*. Therefore the subspecies *Glomerismarginataponentina* Verhoeff, 1911 and *Glomerismarginataleridana* Attems, 1927 are synonymised under the nominal species.

Only initial new naming acts are listed. Due to the numerous mentions of *G.marginata* in the literature, a comprehensive list of all citations is not provided.

### *Glomerismarginata* (Villers, 1789)

*Oniscusmarginatus* Villers, 1789: 187 (first description, type locality “Gallia australiori” – south France)

*Glomerismarginata* – Latreille, 1802: 66 (placing the taxon in the genus *Glomeris*)

### Synonyms

*Juluslimbatus* Olivier, 1792: 414 = *Glomerislimbatus* (Latreille, 1802: 66)

*Armadillomarginalis* Culver, 1792: 30, fig. 23–25, new synonym

*Oniscuszonatus* Panzer, 1793: Heft 9, chapter 25

*Julusoniscoides* Steward, 1802, chapter V: 307

Glomerismarginatavar.lucida Latzel, 1890: 365 and 367

*Glomerisperplexa* Latzel, 1895: 7 and 11, new synonym

*Glomerisconnexaperplexa* Verhoeff, 1906: 152

*Glomerisconnexaperplexa* aberr. *rhenanorum* Verhoeff, 1906: 152 and 153

Glomerisconnexaperplexavar.rhenana Verhoeff, 1906: 152

*Glomerismarginata* aut. *genuina* Verhoeff, 1911: 121

Glomerismarginatavar.marginata Verhoeff, 1911: 121

Glomerismarginatavar.perplexa Verhoeff, 1911: 121

*Glomerismarginataponentina* Verhoeff, 1911: 122, new synonym

*Glomerismarginataleridana* Attems, 1927: 250, new synonym

The description of *Oniscusvariegatus* Villers, 1789: 188, fig. 16 (“niger, segmentis corporis nigris, albo marginatis …” - black, the segments of the body black, white framed) also perfectly fits *G.marginata* and therefore could potentially be treated as a junior synonym of it. However, with the case 2909 of the International Commission on Zoological Nomenclature it was already treated as a senior synonym of *Armadillidiumvulgare* Latreille, 1804 and placed on the Official Index of Rejected and Invalid Species Names in Zoology (Lehtinen and Holthuis 1995, ICZN 1998).

## Analysis software used in this study

BIOEDIT 7.2.5: http://www.mbio.ncsu.edu/bioedit/bioedit.html

DNASP 5.10.1: http://www.ub.edu/dnasp

ESTIMATES 9.1.0: http://viceroy.eeb.uconn.edu/estimates

FIGTREE 1.4.2: http://tree.bio.ed.ac.uk/software/figtree

GLOBALMAPPER 17: http://www.bluemarblegeo.com/products/global-mapper.php

MEGA 7.14 GUI: http://www.megasoftware.net

MICROSOFT EXCEL 2013: http://www.microsoftstore.com

POPART 1.7: http://popart.otago.ac.nz

## Supplementary materials

Supplementary material 1 Complete sequences dataset Data type: FASTA format. Explanation note: Complete sequences dataset of all specimens of this study in format FASTA. This dataset is made available under the Open Database License (http://opendatacommons.org/licenses/odbl/1.0/). The Open Database License (ODbL) is a license agreement intended to allow users to freely share, modify, and use this Dataset while maintaining this same freedom for others, provided that the original source and author(s) are credited.Hans S. Reip, Thomas Wesener

Supplementary material 2 P-distance matrix Data type: Microsoft Excel Worksheet (.xls). Explanation note: P-distance matrix over all specimens as EXCEL-file. Export from MEGA7. This dataset is made available under the Open Database License (http://opendatacommons.org/licenses/odbl/1.0/). The Open Database License (ODbL) is a license agreement intended to allow users to freely share, modify, and use this Dataset while maintaining this same freedom for others, provided that the original source and author(s) are credited.Hans S. Reip, Thomas Wesener

Supplementary material 3 P-distance – geographical distance Data type: Microsoft Excel Worksheet (.xls). Explanation note: P-distance – geographical distance table of all *G.marginata* specimens as EXCEL-file. This dataset is made available under the Open Database License (http://opendatacommons.org/licenses/odbl/1.0/). The Open Database License (ODbL) is a license agreement intended to allow users to freely share, modify, and use this Dataset while maintaining this same freedom for others, provided that the original source and author(s) are credited.Hans S. Reip, Thomas Wesener

Supplementary material 4 POPART-data Data type: NEXUS format. Explanation note: POPART-data file of haplotypes in format NEXUS. This dataset is made available under the Open Database License (http://opendatacommons.org/licenses/odbl/1.0/). The Open Database License (ODbL) is a license agreement intended to allow users to freely share, modify, and use this Dataset while maintaining this same freedom for others, provided that the original source and author(s) are credited.Hans S. Reip, Thomas Wesener

Supplementary material 5 ESTIMATES-data Data type: Text Document (.txt). Explanation note: ESTIMATES-data file of haplotypes as text file. This dataset is made available under the Open Database License (http://opendatacommons.org/licenses/odbl/1.0/). The Open Database License (ODbL) is a license agreement intended to allow users to freely share, modify, and use this Dataset while maintaining this same freedom for others, provided that the original source and author(s) are credited.Hans S. Reip, Thomas Wesener
